# Strategies and challenges for cataract surgery in Brazil

**DOI:** 10.5935/0004-2749.2024-1018

**Published:** 2025-06-24

**Authors:** Newton Kara-Junior, Silvana Rossi

**Affiliations:** Department of Ophthalmology, University of São Paulo, São Paulo, Brazil

Cataracts are a current global health problem that cause blindness and low vision. Kara-Junior N and Rossi S investigated challenges in accessing quality medical care for patients with cataracts, focusing on the availability of surgical treatment and the effect of various state policies on the annual number of cataract surgeries performed in Brazil.

Their studies have scientific and practical value, as they can help formulate more effective policies to combat cataract-related blindness by evaluating the prevalence of visual impairment due to cataracts and the number of cataract surgeries performed^(1^-^4)^.

This is a detailed analysis of the effectiveness of various control initiatives and national programs, depending on the funding sources of hospitals performing cataract surgeries. The authors examined in detail the prevalence of blindness and low vision caused by cataracts in Brazil and the annual number of surgeries performed.

The authors indicate that the incentive to increase the number of surgeries is the provision of unlimited federal funding and direct contracts with private companies through state tenders for performing surgeries. Nonetheless, the most effective strategy was the parliamentary amendments directed to specific municipalities through social health organizations (OSS), as 860,000 cataract surgeries were performed in 2022, reducing the overall number of people suffering from cataract blindness (Table 1)^(1^-^4)^.

**Table 1:** Number of cataract surgeries performed by the Public Health System in Brazil between 2000 and 2022^(1^-^3)^.

Number of cataract surgeries performed in Brazil	
Year	Frequency
2000	230.638
2001	246.876
2002	305.660
2003	431.408
2004	282.760
2005	283.959
2006	189.811
2007	234.045
2008	255.120
2009	320.431
2010	356.088
2011	435.778
2012	461.473
2013	528.677
2014	564.859
2015	473.585
2016	456.297
2017	490.015
2018	563.879
2019	662.536
2020	406.558
2021	640.408
2022	862.713

We considered that the most important message from these studies is that in 2022, Brazil finally managed to perform a sufficient number of cataract surgeries for the needs of its population, emphasizing that financing was the only factor that limited the progression in the number of surgeries. There was no lack of doctors or medical structure.

We ponder that the next steps should be as follows:

- Guarantee the sustainability of financing, as community campaigns and parliamentary amendments are temporary solutions. Creating permanent financing policies for surgeries is essential;
- Improve the quality of surgeries, as surgical campaigns are associated with an increased risk for infections. The results of the National Cataract Campaign (CNC), which lasted until 2006, revealed the availability of hospitals and doctors in sufficient quantity to absorb a large number of surgeries in the routine of public services.

## Brazilian need for cataract surgeries

According to the World Health Organization (WHO), Brazil requires at least 600,000 surgeries to meet the annual demand for the treatment of cataract-induced blindness. Considering that approximately 25% of the population has access to private healthcare, 450,000 surgeries would be required to be performed annually within the Unified Health System (SUS). The WHO has also determined that if the goal is to prevent “economic blindness”, which refers to the compulsion of individuals to leave the workforce due to cataract-related visual impairment, one million procedures would be required annually, corresponding to 750,000 surgeries to be performed by the SUS. Remarkably, this estimate does not include visual rehabilitation for cataract-related blindness cases that had accumulated over time^(1)^.

## Cataract project

In 1986, the Cataract Project was developed by Dr Newton Kara-José as the first organized initiative in Brazil to prevent cataract-related blindness. This initiative used an active screening approach, whereby individuals who were blind due to cataracts were diagnosed and subsequently scheduled for surgery at university hospitals as part of their routine operations^(5^-^8)^.

## National Cataract Campaign (CNC)

Analysis of the SUS database over the years showed that 130,000 cataract surgeries were performed annua-lly until the early 2000s. The CNC was initiated by the Ministry of Health in 2001, with the federal government providing “extra budget” funding to increase the number of surgeries^(7^-^8)^.

The Cataract Project expanded its national reach through the CNC, and all patients diagnosed with cataract-related blindness were operated within the standard operating procedures of public hospitals nationwide. The guaranteed funding stimulated an increase in the surgical capacity of hospitals^(7)^.

In 2003, the SUS performed 431,000 cataract surgeries (Table 1), emphasizing the importance of funding in the prevention of cataract-related blindness in Brazil^(1^-^3)^.

## New public policies

The Ministry of Health discontinued the CNC in 2006. The number of cataract surgeries performed by the SUS decreased in the subsequent years, reaching 430,000 annual procedures again only in 2011, when a new public policy was implemented. To perform the surgeries, this policy involved the government directly contracting private companies through public tenders. In 2012, the SUS performed 461,000 procedures, and for the first time, the minimum number of surgeries required to prevent the accumulation of cataract-related blindness was achieved (Table 1)^(7)^.

The rate of cataract surgeries performed by the Brazilian SUS increased between 2010 and 2019. The increase was linear until 2014, after which a period of decline was observed. In 2017, a new sustained progression was observed (Table 1). In 2014, Brazil experienced an economic crisis that resulted in the suspension of several public policies aimed at promoting cataract surgery^(1^-^3)^.

Parliamentary amendments, which allocated resources to specific municipalities through OSS, were one of the primary sources of funding, alongside actions from the Ministry of Health for cataract surgeries within the SUS in the early 2020s. This strategy to combat blindness proved effective in terms of the number of surgeries performed, with approximately 860,000 cataract surgeries being performed in 2022 (Table 1). Nevertheless, it exhibited the following two drawbacks: the specific municipalities in which investments were made were determined by the parliamentarians, frequently without considering national public health priorities, and a large volume of surgeries were performed over a short period in adapted facilities^(1^-^4)^.

Currently, surgical campaigns are structured to perform procedures in large volumes. These campaigns do not resemble the initial diagnostic campaign proposal, which aimed to identify and distribute confirmed cases for surgery within the regular routines of standard hospitals. In these surgical campaigns, hundreds of surgeries are performed by a small number of doctors in a short period of time in ad hoc operating rooms. Perhaps unsurprisingly, hundreds of patients experienced postoperative complications, with several of them losing their vision due to serial infections^(8)^.

Although, for the first time in history, the SUS has successfully performed an adequate number of cataract surgeries to address the accumulation of new cases and reduce the number of people blinded by cataracts, the appropriateness of the methods used remains skeptical.

It is important to emphasize that the absence of consistency in the number of surgeries performed makes it challenging to establish and maintain local surgical capacity. Government policies must be consistent to ensure a continuous and secure flow of access to treatment, avoiding fluctuations, especially in regions with a predominance of low-income individuals and limited health resources^(9)^.

When examining the public policies adopted in recent decades for visual rehabilitation due to cataracts, the current strategy, which is based on partnerships between public and private sectors, is as effective as the CNC performed in the early 2000s. If the campaign had been sustained, it might have been improved over the years, eventually achieving comparable outcomes.

There are three primary distinctions between the two public policies, viz., the establishment and maintenance of local surgical capacity, improved safety of surgeries performed in suitable facilities, and development of human resources, as the CNC predominantly performed surgeries in teaching hospitals, which contributed to the training of surgeons. A hybrid model would be most suitable for Brazil, as it would capitalize on the esta-blished surgical capacity of public teaching hospitals and the flexibility of private healthcare facilities.

The history of cataract surgeries indicates that the most secure approach would be to adhere to the initial proposal of performing screening campaigns for patients with cataract, scheduling surgeries as part of the routine of regional hospitals to avoid unnecessary strain on surgeons, and implementing safety protocols. In this scenario, logistics would focus on facilitating the transport of patients and expanding the surgical capacity of regional hospitals, engaging them in the project elements that the CNC demonstrated to be feasible.
